# Supplementary material for: Skeletal Muscle Cells Derived from Induced Pluripotent Stem Cells: A Platform for Limb Girdle Muscular Dystrophies
Source: Biomedicines. 2022 Jun 16;10(6):1428. doi: 10.3390/biomedicines10061428 (PMC9220148; doi:10.3390/biomedicines10061428)
Supplement: Supplementary file 1 [file biomedicines-10-01428-s001.zip › biomedicines-1725818-supplementary/Supplementary Figures and Tables.pdf]

Figure S1

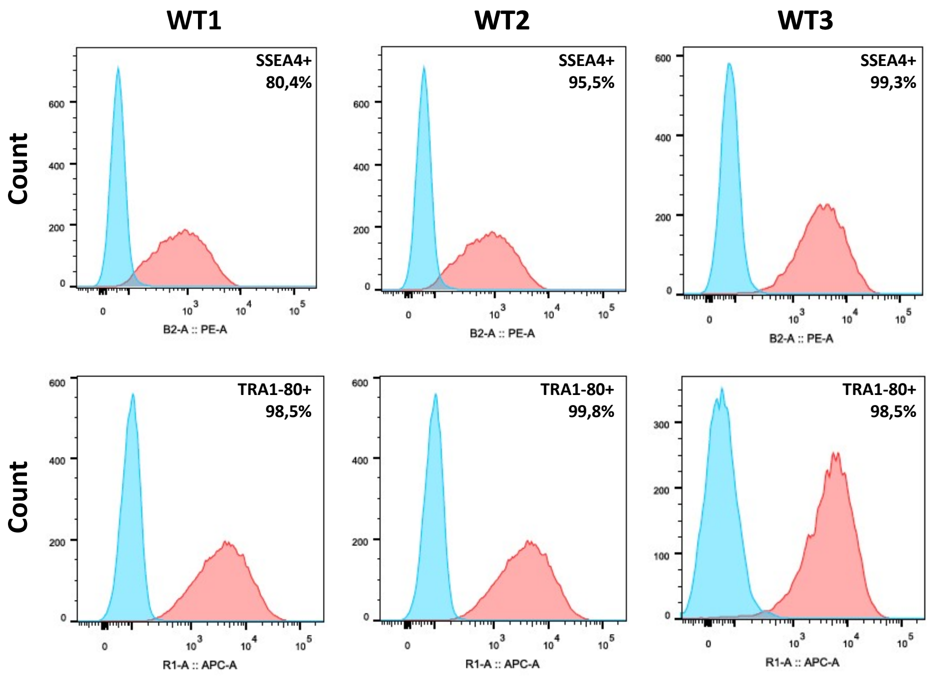

WT1
WT2
WT3
Count
SSEA4+
80,4%
SSEA4+
95,5%
SSEA4+
99,3%
B2-A :: PE-A
Count
TRA1-80+
98,5%
TRA1-80+
99,8%
TRA1-80+
98,5%
R1-A :: APC-A

Figure S2

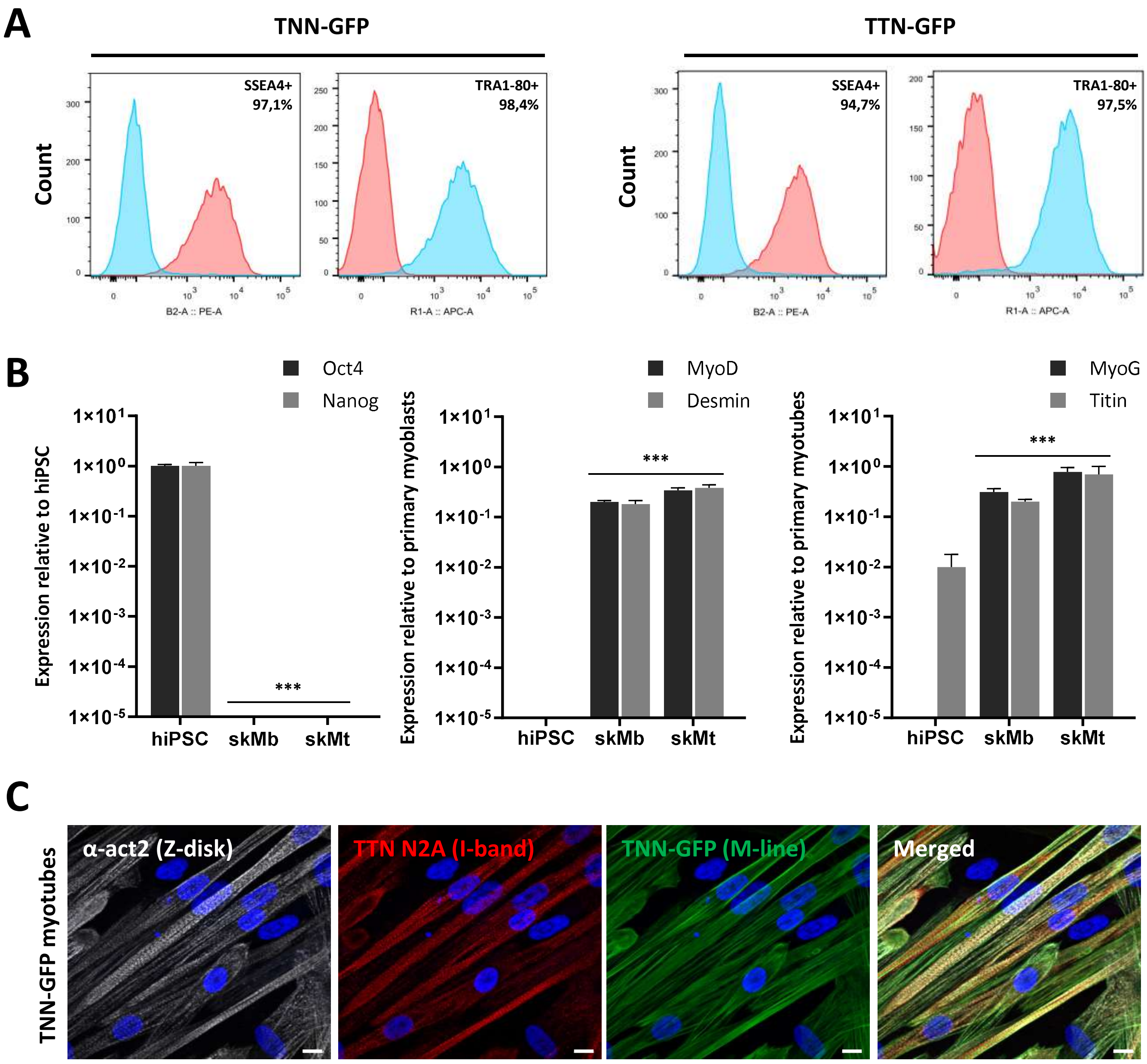
A
TNN-GFP
TTN-GFP
SSEA4+
97,1%
TRA1-80+
98,4%
SSEA4+
94,7%
TRA1-80+
97,5%
Count
B2-A :: PE-A
R1-A :: APC-A
B
Oct4
Nanog
MyoD
Desmin
MyoG
Titin
Expression relative to hiPSC
Expression relative to primary myoblasts
Expression relative to primary myotubes
***
hiPSC
skMb
skMt
C
TNN-GFP myotubes
α-act2 (Z-disk)
TTN N2A (I-band)
TNN-GFP (M-line)
Merged

Figure S3

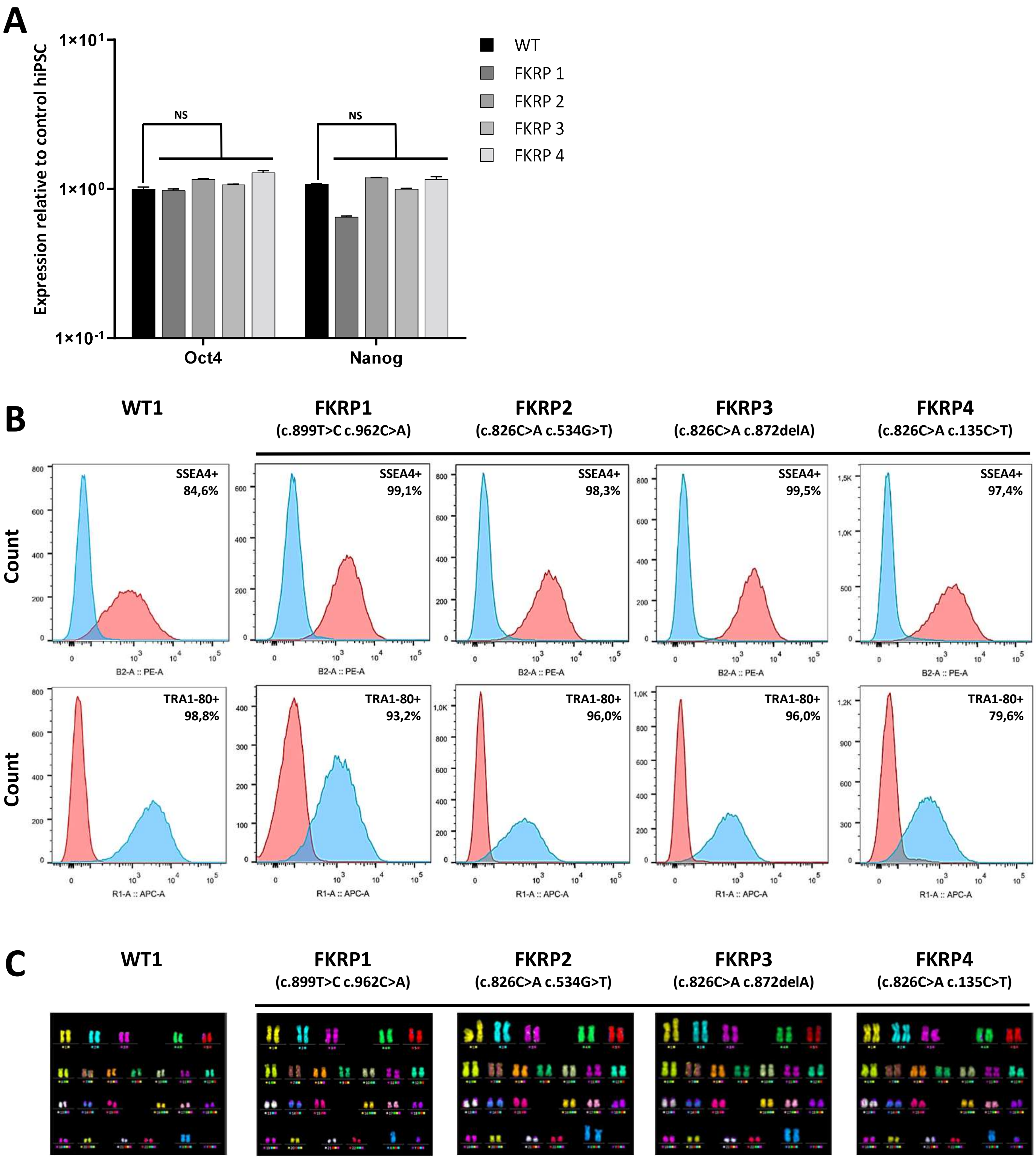
A
Expression relative to control hiPSC
1×10¹
1×10⁰
1×10⁻¹
NS
NS
Oct4
Nanog
WT
FKRP 1
FKRP 2
FKRP 3
FKRP 4
B
WT1
FKRP1
(c.899T>C c.962C>A)
FKRP2
(c.826C>A c.534G>T)
FKRP3
(c.826C>A c.872delA)
FKRP4
(c.826C>A c.135C>T)
Count
SSEA4+
84,6%
SSEA4+
99,1%
SSEA4+
98,3%
SSEA4+
99,5%
SSEA4+
97,4%
B2-A :: PE-A
Count
TRA1-80+
98,8%
TRA1-80+
93,2%
TRA1-80+
96,0%
TRA1-80+
96,0%
TRA1-80+
79,6%
R1-A :: APC-A
C
WT1
FKRP1
(c.899T>C c.962C>A)
FKRP2
(c.826C>A c.534G>T)
FKRP3
(c.826C>A c.872delA)
FKRP4
(c.826C>A c.135C>T)

Figure S4

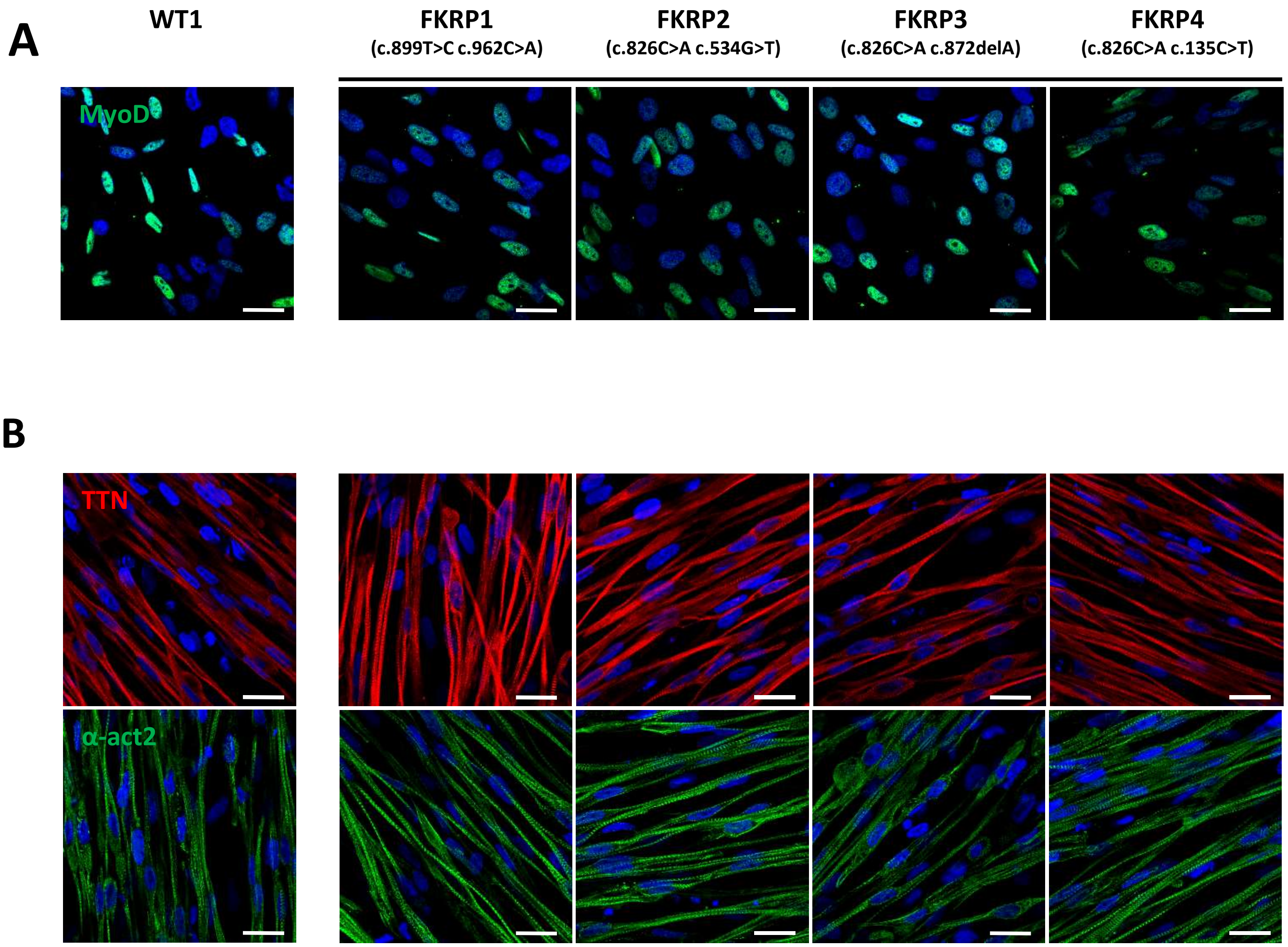
A
WT1
FKRP1
(c.899T>C c.962C>A)
FKRP2
(c.826C>A c.534G>T)
FKRP3
(c.826C>A c.872delA)
FKRP4
(c.826C>A c.135C>T)
MyoD
B
TTN
α-act2

Table S1

Table S1. Classification and nomenclature of LGMD.

New nomenclature	Old nomenclature	Gene	Protein
LGMD D1	LGMD 1D	DNAJB6	DNAJB6, DnaJ heat shock protein family member B6
LGMD D2	LGMD 1F	TNPO3	TNPO3, Transportin 3
LGMD D3	LGMD 1G	HNRNPDL	HNRnPDL, Heterogeneous nuclear ribonucleopreotein D like
LGMD D4 / R1	LGMD 1I / 2A	CAPN3	Calpain 3
LGMD D5 / R22	Bethlem myopathy dominant / recessive	COL6A1, COL6A2, COL6A3	Collagen 6
LGMD R2	LGMD 2B	DYSF	Dysferlin
LGMD R3	LGMD 2D	SGCA	α-sarcoglycan
LGMD R4	LGMD 2E	SGCB	β-sarcoglycan
LGMD R5	LGMD 2C	SGCG	γ-sarcoglycan
LGMD R6	LGMD 2F	SGCD	δ-sarcoglycan
LGMD R7	LGMD 2G	TCAP	Telethonin
LGMD R8	LGMD 2H	TRIM32	TRIM32, Tripartite motif containing protein 32
LGMD R9	LGMD 2I	FKRP	FKRP, Fukutin-related protein
LGMD R10	LGMD 2J	TTN	Titin
LGMD R11	LGMD 2K	POMT1	POMT1, Protein O-mannosyl-transferase 1
LGMD R12	LGMD 2L	ANO5	Anoctamin 5
LGMD R13	LGMD 2M	FKTN	Fukutin
LGMD R14	LGMD 2N	POMT2	POMT2, Protein O-mannosyl-transferase 2
LGMD R15	LGMD 2O	POMGNT1	POMGNT1, Protein O-linked mannose N-acetylglucosaminyltransferase 1 (beta 1,2-)
LGMD R16	LGMD 2P	DAG1	Dystroglycan 1
LGMD R17	LGMD 2Q	PLEC	Plectin
LGMD R18	LGMD 2S	TRAPPC11	TRAPPC11, Trafficking protein particle complex subunit 11
LGMD R19	LGMD 2T	GMPPB	GMPPB, GDP-mannose pyrophosphorylase
LGMD R20	LGMD 2U	ISPD	ISPD, 2-C-methyl-D-erythritol 4-phosphate cytidylyltransferase
LGMD R21	LGMD 2Z	POGLUT1	POGLUT1, O-transferase 1 protein
LGMD R23	Laminin α2-related muscular dystrophy	LAMA2	Laminin α2
LGMD R24	POMGNT2-related muscular dystrophy	POMGNT2	POMGNT2, Protein O-Linked Mannose N-Acetylglucosaminyltransferase 2 (Beta 1,4-)

Table S2

Table S2. List of primers.

LGMD	Gene	Forward	Reverse	Taqman MGB probe
LGMD D1	DNAJB6	CCCTTTTGAGGACTTCTTTGGG	AGAGAAAAACGACCCCGTCC	
LGMD D2	TNPO3	AAACCTCCGTTGTGGCAGAG	CACACCCCCAAACAGGAACT	
LGMD D3	HNRNPDL	GATCCCAAAAGGGCCAAAGC	GTATCCGGGCTCAATCCACC	
LGMD D4 / R1	CAPN3	Assay Hs00544977 (Applied Biosystem)		
LGMD D5 / R22	COL6A1,<br>COL6A2,<br>COL6A3	CCATTGGCCTCTGTCTCGTT<br>GTTCCACGAGAAGCACGAGA<br>ACAGTGACGAGGTGGAGATCA	TACTCTCTGAGGTCGGGGTC<br>GAGAACAGCGTCATGTTGCG<br>GATAGCGCAGTCGGTGTAGG	
LGMD R2	DYSF	Assay Hs01002513 (Applied Biosystem)		
LGMD R3	SGCA	TGCTGGCCTATGTCATGTGC	TCTGGATGTCGGAGGTAGCC	CGGGAGGGAAGGCTGAAGAG AGACC
LGMD R4	SGCB	CCCGCCTACCCAGTTCCT	TGTAGCGTACCCAGTCACCACTA	CAGTGGAGACCAGTTGG
LGMD R5	SGCG	TGGACTCATCTCTGCTGCTACAA	CTTCTGAGTTGCGCGCATT	CAACCCAGAATGTGACTGT
LGMD R6	SGCD	GGGCACAGTGTTCCCTAAATCT	GGGACTCCAACCTTAGTTCTTTGA	TAGAAACACCTAATGTCAGGG CAGACC
LGMD R7	TCAP	TGAGTTCCCAAAGGGAGGGT	CTGCCTAGAGCGTTCCAGAT	
LGMD R8	TRIM32	TGGGCATAGCCCTAACTCCT	GAAGGGAAGTTGGCTTGGGA	
LGMD R9	FKRP	Assay Hs00748199 (Applied Biosystem)		
LGMD R10	TTN	Assay Hs00399225 (Applied Biosystem)		
LGMD R11	POMT1	GGGTGGAGTTTGATCTGGCA	CAATGACATGCAAGCCCCAG	
LGMD R12	ANO5	Assay Hs01381106 (Applied Biosystem)		
LGMD R13	FKTN	TGAGAGTAAAGATCCCCGGC	CATGAGTGGCCAGTTTGCAG	
LGMD R14	POMT2	TGGTGCTTTAGGGGTCAAGT	ACCAAAGGTCTGCAATGGTG	
LGMD R15	POMGNT1	ACACAGAGCTGAACCGTCG	GGCTGAACTCGATGGGTGT	
LGMD R16	DAG1	Assay Hs00189308 (Applied Biosystem)		
LGMD R17	PLEC	CGCCTTCTGCCACTGAAAGG	AATGCCCTTCTTGTCTCCTCC	
LGMD R18	TRAPPC11	AGCATCCCAAGTGTGACTGG	GAGACACATGGGGGATTCGG	
LGMD R19	GMPPB	TTCACCGGTTCGTGGAGAAG	TGTCCATCCAGAAGCCCTGT	
LGMD R20	ISPD	AGGCCCTGGAGAGAGTATGTT	CTTCGACCAGTGAGATGCGT	
LGMD R21	POGLUT1	GGTCTAAGGAAGCGGTAGCC	AGTAGGGCATGAAGGCACAA	
LGMD R23	LAMA2	CGGCCTGGAAACTGGATTTTG	GTTAGGCACTCCGTGTCTGT	
LGMD R24	POMGNT2	GAGTCCAGGCTTCAGGCTTTCA	GAGATGACGGCCACTGGGAA	

Table S3

Table S3. List of antibodies.

Protein	Supplier	Reference	Dilution
DNAJB6	Proteintech	117107-1-AP	1/100
TNPO3	Abcam	ab54323	1/100
HNRnNPDL	Thermofisher	PA5-63354	1/100
Calpain 3	Santa Cruz	Sc-365277	1/100
COL6A1,<br>COL6A2,<br>COL6A3	Proteintech<br>Santa Cruz<br>Santa Cruz	17023-1-AP<br>sc-374566<br>Sc-515335	1/100
Dysferlin	Abcam	Ab124684	1/100
α-sarcoglycan	Novocastra	A-SARC-L-CE	1/100
β-sarcoglycan	Novocastra	NCL-L-b-SARC	1/100
γ-sarcoglycan	Novocastra	NCL-g-SARC	1/100
δ-sarcoglycan	Novocastra	NCL-d-SARC	1/100
Telethonin	Santa cruz	sc-25327	1/100
TRIM32	Abnova	H00022954-M09	1/100
FKRP	Généthon	Homemade	1/1000
Titin	US Biological	T5650	1/100
POMT1	Novus Biological	NBP2-57200	1/100
Anoctamin 5	Covalab	pab0597-0	1/200
Fukutin	Abcam	ab113233	1/300
POMT2	Santa Cruz	sc-393487	1/100
POMGnt1	Santa cruz	Sc-130459	1/400
Dystroglycan 1	DSHB	VIA41-s	1/10
Plectin	Abcam	ab192706	1/400
TRAPPCC11	Interchim	Orb51790	1/100
GMMPPB	Proteitech	15094-1-AP	1/100
ISPD	Abcam	ab222793	1/400
POGLUT1 (=KTELC1)	Proteintech	24409-1-AP	1/100
Laminin α2	Abcam	ab11576	1/100
POMGnt2	LifeSpan BioSciences	LS-C335679	1/200
α-actinin2	Thermofisher	701914	1/100
CD56-PE	Miltenyi Biotec	130-090-755	1/100
Desmin	R&D	AF3844	1/200
MF20 (MHC)	DSHB	3ea	1/100
MyoD	Dako	M3512	1/50
MyoG	DSHB	F5D-c	1/50
Nanog	Cell signaling	4903-s	1/100
Oct4	Cell signaling	2840-s	1/100
SSEA-3-PE	Millipore	330312	1/10
TRA-1-81-AF647	Covance	560124	1/25

* Without permeabilization step
